# #fitspiration: a comparison of the sport-related social media usage and its impact on body image in young adults

**DOI:** 10.1186/s40359-022-01027-9

**Published:** 2022-12-27

**Authors:** Kristina Klier, Tessa Rommerskirchen, Klara Brixius

**Affiliations:** 1 Institut für Sportwissenschaft, Universität der Bundeswehr München, Neubiberg, Germany; 2 Institut für Kreislaufforschung und Sportmedizin, Deutsche Sporthochschule Köln, Cologne, Germany

**Keywords:** Body image, Digital natives, Fitness trend, Fitspiration, Social media

## Abstract

**Background:**

Following and posting sport-related content on social media is wide-spread among young people. To date, little is known about the interdependence between sport-related social media use and the thereby perceived personal body image.

**Methods:**

We conducted an online survey (*N* = 285) to examine how social media influences the sport-related body image.

**Results:**

In general, social media are frequently used for sport (*n* = 136, 47.7%). Resistance training correlated significantly with several motives of sport-related use of social media, and thus, represents the strong online presence of athletic sports. Less correlations could be found in team or other sports. Regarding the perception of body image, it was found that the group of rejecting (negative) body image significantly correlated with the emulation of social media mediated sport-related beauty and body ideals (*r* = 0.63, *p* = 0.001), as well as with increased body dissatisfaction when viewing sport-related posts on social media (*r* = 0.590, *p* = 0.001). Perceived social pressure and comparison were found to be mediators of the prevailing influence of social media usage.

**Conclusions:**

These results reveal the importance of taking a closer look at socially shaped beauty and body ideals, especially in sport-related contents, striving for more educational campaigns such as *Body Positivity* and, above all, filtering information. Finally, future research is needed to gain deeper insight into young persons’ usage behavior of social media and its impact on the individual’s body image.

*Trial Registration* The study was conducted according to the guidelines of the Declaration of Helsinki, and approved by the Ethics Committee of University of the Federal Armed Forces Munich, Germany (01/24/2022).

## Introduction

Nowadays, almost all young people use social media [1]. Known as *Digital Natives*, they grew up with the internet and social media [2]. Platforms such as Instagram, Facebook or Twitter are widely spread, and so-called ‘influencers’ constantly propose must-haves, what to eat and drink, what to wear or how to do sport. For the young, digital generation health and fitness content play an important role. On social media they are literally looking for nutrition, health and fitness information whilst the focus often is on physical improvement. In line, the findings of a recent study by Cataldo et al. [3] have revealed that, in particular during the Covid-19 pandemic, the social and fitness-related use of social media is significantly increased. Aside, the discussion about the socially embossed body ideal is omnipresent. Especially social media offer platforms for presenting socially standardized body ideals [4]. Moreover, the plurality of influencers presents nearly 24/7 their healthy and sporty lifestyle [5]. That is why sport and fitness take on a new meaning by social media. The ideal body demanded by society today corresponds to a slim, well-trained body, i.e., an athlete body [6]. Nevertheless, the sporty body staging and representations of a healthy lifestyle can also have few risks. Unfiltered knowledge and unrealistic representations can cause negative effects [7]. In line, recent debates on social media point out and criticize the unscientific or irresponsible messages that are increasingly spread. Subjective opinions and experiences that are shared are to some extent responsible for suspicious trends [8]. One of the current trends, for example, relates to so-called ‘Fitspiration’ content and images that, on the one hand, should increase the daily motivation, and on the other hand, inspire the followers to achieve the desired fit body [9]. Content relating to the main topics of health, fitness, nutrition and lifestyle is tagged by the hashtag *#fitspiration*. The (fitness-)influencers not only present a perfect body in their posts, but also often promote branded clothing and other products related to nutrition and exercise [10]. Fitspiration-related content such as ‘before and after’ images, sugar-free or low-carb food, body tuning workouts and so on are intended to show the ideal of health and fitness. A trend that does not seem to be negative at first sight, but there are often non-scientific and inaccurate statements behind Fitspiration-related posts. In particular, they propagate certain extremes, and thus, depict unrealistic body ideals. This trend is mainly followed by women, who in turn are also mostly affected by possible negative effects such as eating disorders or depression [11–13]. Usually, a healthy approach to the own body considers a positive self-esteem which in turn is responsible for how well socially shaped body ideals can be reflected. I.e., a healthy, positive approach can prevent negative effects on mental and physical health. If a person appears dissatisfied with his or her own body image, he/she often experiences negative feelings about the body shape, for example, in relation to the body weight. Likewise, Cataldo et al. [14] have reviewed that especially adolescent females but also in general (young) people with low self-esteem or high anxiety might negatively be influenced by Fitspiration content which in turn can lead to compulsive exercising or excessive control of eating habits. Besides such psychopathological risks, Barron et al. [15] have shown that viewing Fitspiration images overall promotes a decrease in body satisfaction and appreciation in both gender whilst viewing self-compassion images supports a positive body approach and seems to have a buffering effect.

Studies indicate that the influence of the internet and especially of social media to convey body ideals is higher today than that of television and print media [16]. As there is constant online-confrontation with representations of unattainable body ideals, the possibility of comparison is much higher than before [17]. Hence, the body and its appearance are becoming more and more important, and it is becoming increasingly important for society to live up to the contemporary ideal of the body. Social media are also responsible for shaping specific body ideals. A current guiding theme is the slim and athletic body: The *Athletic Ideal*, a body ideal, which emerged from a new understanding respectively a new level of perfection prevailing on social media as well as in wide-spread fitness magazines. This ideal arose out of a growing interest in health and sports. It corresponds to a very slim, but still muscular person [18]. At first sight, the *Athletic Ideal* presents a positive, healthy lifestyle, but to reach that a strict diet and hard training are needed. The message behind the *Athletic Ideal* conveys a happy and fulfilling life. In particular, young people expect to be able to reach this ideal quickly and easily as this is repeatedly suggested by fitness-influencers. Studies have shown that the lifestyle presented can have a negative impact on person’s body image comparable to the one of the *Thin Ideal*, ranging from a distorted perception of the body to a compulsive need for movement [19]. As of late, a movement known as *Body Positivity* is gathering more and more followers [10, 20]. It presents alternative body ideals in which the appreciation of one's own body is the focus. The commonly trivialized role of *Thinspiration* and *Fitspiration* content is responsible for the glorification of extreme thinness as well as for the ideal conception of body image [21].

Although the young adults grew up digitally and it is well known that social media can hold various danger, even in sporty representations, most users follow and implement the content without questioning it. This particularly loads since the young people’s self-confidence and self-esteem are not entirely developed yet but are still rising which in turn means a vulnerable body image. In addition, neither the users’ behavior nor the impact and reliability of health- and fitness-related content have been examined comprehensively. Therefore, the present study aimed to investigate the sport-related use of social media and the resulting influence on body image in a young, healthy sample. We hypothesized that, on the one hand, most of the respondents regularly use social media to find inspiration and motivation for a sporty and healthy life. On the other hand, persons in athletic sports, as well as especially women, might be more influenced and will report a greater impact respectively less self-consciousness regarding their body image.

## Methods

### Participants

In total, 285 participants, of them 101 (35.4%) males and 184 (64.6%) females, took part in the study. To be included, participants needed to be between 18–40 years old and use social media daily. Persons who were older than 40 years (i.e., who were not grown up digitally or not used to a daily digital approach), did not have a smartphone and were not active on social media were excluded from the study. We then divided the total sample into two age groups: The age group 18–25 years included 230 participants (80.7%; 74 males, 156 females), and the age group 26–35 years incorporated 55 participants (19.3%; 27 males, 28 females).

### Study procedure

The study was conducted using a common online survey tool that met the university’s ethics and privacy policy which in turn is consistent with the European data privacy act. For investigation, we used the standardized body image questionnaire (FKB-20) by Clement and Löwe [22] and added further questions about participants’ sportive background and (sport-related) social media usage behavior. We shared the link via email and social media with the university’s network as well as local sports and fitness clubs. Finally, the questionnaire was online available for two weeks. The study was approved by the local ethics committee and participants provided informed written consent prior to the study.

### Material

The used online questionnaire comprised five parts. The first section asked for sociodemographic data such as sex and age. The second section consisted of sport-related questions such as the frequency of exercise per week, the duration of the trainings, the type of sport, the forms of organization and the motives for being physical active. The last part of these single and multiple answer questions was related to the usage behavior of social media. Here, we included questions about the duration and type of use, as well as the usage motives with a focus on sport-related motives. Questions were, for example, *“Which of the following social media do you usually use?”* (Facebook, Twitter, Instagram, LinkedIn, YouTube, Other [could be specified in a text field]), *“What do you usually use social media for?”* (Maintain contacts, Establish contacts, Exchange of opinions and experiences, Source of information, Self-presentation, Sports (e.g., information, motivation), Pastime/Entertainment, Other [could be specified in a text field]), *“To what extent do you use social media for sports?”* (Training motivation, Training Tips/Information, Sports inspiration, Nutrition tips, Communication about sports, Sportive self-staging (e.g., sport postings), Marketing purposes (e.g., for club), Following athletes in public life (e.g., fan communication), Other [could be specified in a text field]), *“Are you familiar with sports-related beauty and body ideals (e.g., fitness lifestyle, Athletic ideal, *etc*.) on social media platforms?”* (Yes, No, Not applicable). The fourth section was related to the overarching themes of sport, social media and body image and were questioned by using a 5-point Likert scale (1 = *does not apply at all*, 5 = *fully applies*). The section focused on the subjective perception of the participants. Questions were amongst others: *“Have you emulated/Do you emulate a certain sport-related ideal of beauty and body shape?”*, *“How strongly do you feel influenced by social media fitness communities with regard to your appearance/body shape?”*. As rating items, we formulated, for example: *“I actively follow sport-related content on social media.”*, “*I share on social media myself sport-related content.”*, “*When I look at posts on social media, my dissatisfaction with my own body increases.”*, “*I feel social pressure on social media, in the pursuit of the perfect body.”*, *“I often compare my body with the body ideals presented on social media.”*, “*I actively look for confirmation of my own body image on social media.”.* The same Likert scaling was used in the fifth section of the questionnaire, which was the standardized and normed body image questionnaire by Clement and Löwe [22]. The *Body Image Questionnaire* (“Fragebogen zum Körperbild”, FKB-20) integrates cognitive, affective and evaluative aspects of body image. It comprises two subscales: *Rejecting Body Image* (RBI; evaluations of an individual’s body image regarding appearance and well-being) and *Vital Body Dynamics* (VBD; energetic and movement related aspects and activities). The negative body evaluation (RBI) summarizes one's own feelings and the external physical appearance. The vital body dynamics scale (VBD) addresses the dimension in which health and fitness are perceived. Thus, VBD scores represent positive body image and RBI scores represent negative body image. We chose this as main assessment tool as the FKB-20 is a valid and reliable inventory (Cronbach’s ɑ_RBI_ = 0.8, Cronbach’s ɑ_VBD_ = 0.9) for assessing the individual body image and related disorders in clinical as well as non-clinical settings.

### Statistical analysis

The statistical analysis was carried out using the Statistic Software SPSS 27. The level of significance was set a priori at α < 0.05. After testing for normality by performing the Shapiro–Wilk test, data was descriptively analyzed. Further, we calculated chi-square and Cramer’s V for normally and ordinally scaled data in order to examine the relationship between sport, social media and body image. If the conditions were met (all cells had an expected frequency greater than five), the Yates-corrected chi-square test (continuity correction) was used for the 2 × 2 tables; if the conditions were not met, we used Fisher's exact test. In addition, a Spearman rank correlation was performed to correlate the interval- and ordinal-scaled data. Results were interpreted according to Cohen [23].

For the evaluation of the FKB-20, the total scale value was calculated by adding each the 10 item points. In comparison to the literature [24], high total scores on the rejecting body image scale imply a negatively perceived body image, and high total scores on the scale for vital body dynamics can be interpreted as positively perceived body image. Except item 5 and 19, that are positively pooled, but refer to the negative scale. Thus, as the sole interpretation of the cumulative value would be inaccurate, we defined the differentiation between positive and negative body image each based on the individual’s mean values plus/minus standard deviation.

## Results

### Descriptive statistics

#### Sportive activities

On average, participants do three to four times per week sport (*n* = 114, 40.0%) with each training lasting 46–60 min (*n* = 84, 29.5%) or even 61–90 min (*n* = 101, 34.4%). Most of them are engaged in athletic (*n* = 104, 36.5%) and endurance sports (*n* = 92, 32.3%). Women tend to exercise less and shorter than men. More details regarding participants sportive background are shown in Table 3 in “Appendix A”.

#### Internet use

**Fig. 1:**
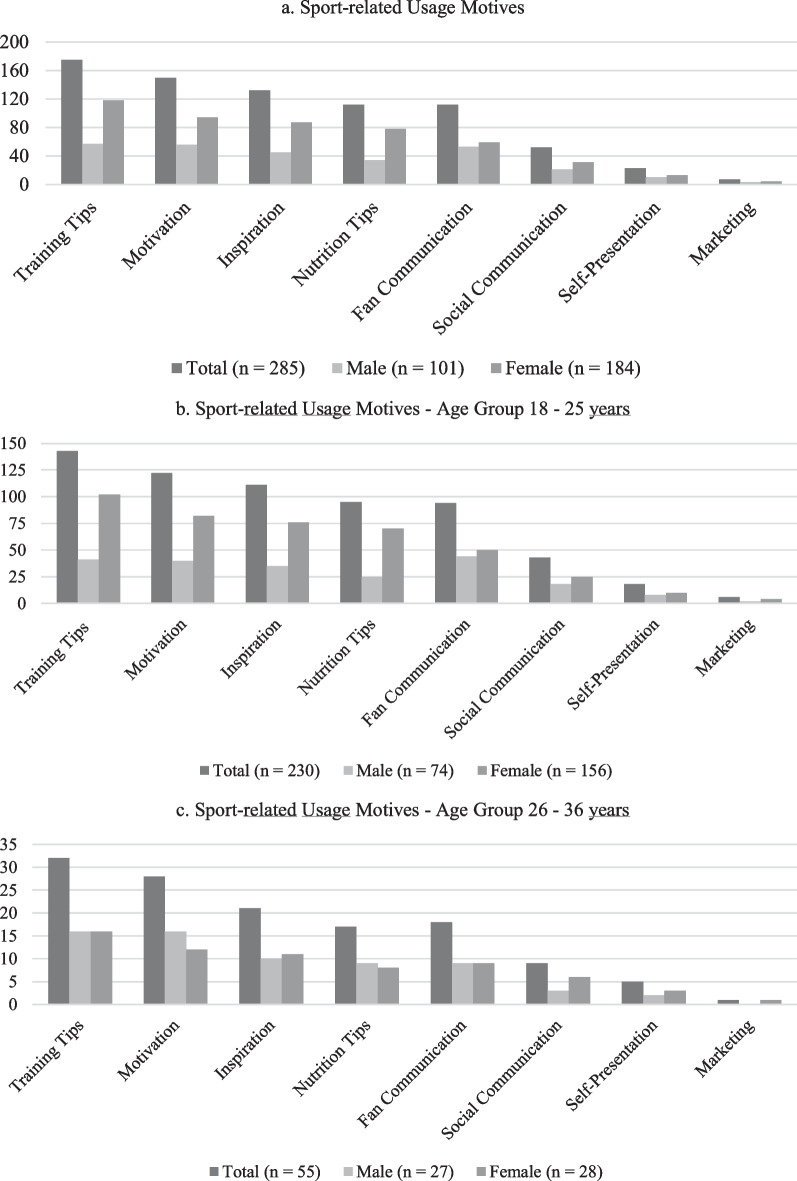
Participants’ usage motives of social media. *Note*. Sport-related usage motives are presented in accordance to the number of participants for the total sample (**a**) and differentiated to the age groups (**b**, **c**)

Respondents spend two to three hours per day using social media (*n* = 166, 58.3%). 92.3% (*n* = 263) are on Instagram, 38.6% (*n* = 110) on Facebook. As multiple answers were possible, it can be assumed that many persons use both platforms similarly. 95.2% (*n* = 219) of Instagram users are in the 18–25 years age group and most of them being female (*n* = 148). Contrarily, only one third of this young age group (*n* = 75, 32.6%) were active on Facebook. When asked what for the individuals use social media, 136 of the totals (47.7%) chose the motive sport. 47.8% (*n* = 110) of 18- to 25-year-olds chose this motive and 47.3% (*n* = 26) of 25- to 35-year-olds. Regarding the sport-related use of social media, again, participants could choose multiple answers. The training motivation motive was chosen by 52.6% (*n* = 150), training tips/training information by 61.4% (*n* = 175), and the inspiration motive by 46.3% (*n* = 132). Each 39.3% (*n* = 112) are looking on social media for nutrition tips and fan communication, followed by, in descending order, the motive of social communication about sport (*n* = 52, 18.2%), sportive self-presentation (*n* = 23, 8.1%), and marketing purposes (*n* = 7, 2.5%). Further differentiation between the age groups and sex are presented in Fig. 1 (“Appendix B”).

#### Body image

When asked whether the participants knew ideals of beauty and body shape, 71.9% (*n* = 205) answered yes and 22.8% (*n* = 65) answered no. Whilst 29.8% (*n* = 85) occasionally emulated a beauty ideal on social media, 26.0% (*n* = 74) have never done it. One-fifth up to one-fourth of women (*n* = 43) often emulate ideals of beauty and body shape whereas only 9.9% (*n* = 10) of men do so. Further, participants feel little to moderately influenced by virtual fitness communities. Men rate this impact mainly neutral while women tend to be influenced negatively. In line, the dissatisfaction towards the own body seems barely to not to be increased when following social media content. However, nearly half of women (*n* = 88, 47.8%) and a little bit more than a third of men (*n* = 36, 35.6%) responded to compare their body with virtual presented body ideals at times. Further, participants do feel barley (*n* = 64, 22.5%) to little (*n* = 60, 21.5%) social pressure to optimize their body in order to suit a presented ideal.

The evaluation of the FKB-20 is illustrated in Table 1. Data are given as means plus/minus standard deviations.

**Table 1:** Classification of participants’ body images

	Collected data	Standard values	Collected data	Standard values
	18–25 years	≤ 24 years	26–36 years	25–34 years
Rejecting body image (RBI)	26.3 ± 6.2	18.3 ± 7.2	26.3 ± 5.7	17.8 ± 6.9
Vital body dynamics (VBD)	33.8 ± 6.0	29.4 ± 6.7	33.6 ± 7.0	38.4 ± 6.9

In order to group the respondents according their evaluated body image, we compared the collected data with the standard values given in the literature [24]. Group 0 was formed from the values within one standard deviation from the mean, group 1 was considered abnormal and was formed from values more than one standard deviation from the normal range, group 2 was defined as extreme and was formed by two standard deviations from the normal range.

Table 2 shows the distribution of the groups according to RBI and VBD. It should be noted that the three classifications do not contain a positive or negative rating, they can only be classified as *normal*, *abnormal* or *extreme*. Most participants could be classified in the normal group (0) of RBI (*n* = 125, 43.9%) and VBD (*n* = 153, 53.7%), and a balanced number of both scales (RBI: *n* = 64, 22.5%; VBD: *n* = 62, 21.6%) were in the abnormal group (1). Notably, with *n* = 41 (14.4%) more than twice as much participants were classified in the extreme group (2) of the RBI scale than the VBD scale (*n* = 15, 5.3%).

**Table 2:** Classification of body image groups

	Rejecting body image (RBI)	Vital body dynamics (VBD)
Standard values	18.3 ± 7.2	29.4 ± 6.7
Group 0 [normal]	11.2–25.4; n = 125	22.7–36.1; n = 153
Group 1 [abnormal]	< 11.2, > 25.4; n = 64	< 22.7, > 36.1; n = 62
Group 2 [extreme]	< 4.1, > 32.5; n = 41	< 16, > 42.4; n = 15

### Inferential statistics

Due to the sub-sample’s power and the better fit to the definition of ‘Digital Natives’, only the 18–25 age group was examined for the further presentation of the results.

#### Sports and social media usage

Table 4 (“Appendix A”) shows the results of the performed chi-square tests of the sports with the given motives as well as the body image groups. The sport-related usage of social media was a significant predictor (*p* = 0.02) in resistance training, in contrast to endurance sport, team sports and other sports. In line, athletic sports correlated significantly with the motive of training motivation (*p* < 0.001), training information (*p* = 0.008) and nutrition tips (*p* ≤ 0.02). The tendency to emulate ideals of beauty and body shape on social media could only be significantly predicted by doing resistance training (*p* < 0.001). Similar to the resistance sports group, a significant correlation of the motive of training motivation (*p* = 0.04) was found for the team sports group. Overall, the effect sizes of the computed chi-square tests were weak to moderate which is why all other sport-related results were not significant.

#### Rejecting body image and vital body dynamics

Although we found no correlations between the sports and RBI as well as VBD, significant correlations between body image groups and participants’ sport-related usage of social media could be determined (see Table 5 in “Appendix A”). The rejecting body image and the emulation of sport-related ideals of beauty and body shape correlated significantly (*p* = 0.001) showing a strong effect (*r* = 0.63). No effect could be found in relation to VBD. This scale weakly correlated with the sport-related time spent on social media off training (*p* = 0.001). Another small, but significant effect was found between the VBD and following sport-related content on social media in general (*r* = 0.20, *p* = 0.002). The VBD also correlated weakly positively with the statement “I share sport-related content myself on Social Media” (*r* = 0.18, *p* = 0.007). In contrast, the RBI showed a strong positive correlation with dissatisfaction towards the own body when viewing sport-related social media posts (*r* = 0.60, *p* = 0.001), and with the feeling of social pressure regarding the perfect body image on social media (*r* = 0.50, *p* = 0.001). At last, also the correlation of the RBI and the comparison with the body ideals on social media resulted in a moderate to strong relation (*r* = 0.48, *p* = 0.001).

## Discussion

The study aimed to examine the relationship between the sport-related use of social media and its influence on body image. As hypothesized, most of the respondents regularly use social media to find inspiration and motivation for a sporty and healthy life. Furthermore, especially individuals engaging in athletic sports seem be more influenced by social media which in turn also correlates with less self-consciousness regarding their body image.

### Social media usage of young adults

It is well known that for young adults, which comprised our set age groups, social media plays an important role [2]. Our findings illustrate the existing enthusiasm for sport (seen for example in the increasing number of people engaging in online sport classes as well as the boom of home-fitness equipment during Covid-19 pandemic [25]), which could be due to the fact that the participants mostly came from sporting institutions. Most of them are engaging in resistance training followed by endurance sport. The less done team sport could be due to current Corona pandemic and its restrictions. As half of men answered to do resistance training, it may be assumed that muscle building and strength training seems to play a greater role in young men, as the masculine ideal of beauty and body shape is oriented on a muscular body [26]. Nevertheless, in women, a little bit more than one third each engage in endurance sport and resistance training. Linked to the sample’s social media usage, this observation supports the findings of Aanesen et al. [27] and should be further investigated. In line, Mayoh and Jones [28] have recently examined in an online survey the fitness-oriented engagement of young males and females on social media. It seems as if a change could be recognized: away from the desired thin body through excessive endurance sports to a muscular, strong body through athletic sports. This change has not been comprehensively investigated by scientific studies, but a connection to the changed use of social media and the posted ideals of beauty and athletic body shape should be discussed [28, 29].

### Motives for sport-related use of social media

The present study was also able to provide an informative insight into the sport-related use of social media. Given the temporal use of 2–3 h per day, our findings are in line with current research [30]. Whilst Facebook is the most popular platform worldwide [31], our respondents mainly use Instagram. This can be confirmed by a recent online survey by the greatest German television channels ARD/ZDF [32]. According whom, Instagram is the most used social network in Germany. In general, social media offer various suggestions and opportunities for the personal usage. Likewise, nearly half of participants chose the use of social media in relation to sport as prevalent usage motive. Tiggemann and Zaccardo [9] have revealed the frequent use of Instagram in relation to sport-related content. Our results showed a significant correlation between the engagement in resistance training and the motive for sport-related use of social media. Resistance training correlated also significantly with motivation and the training information motive. In line, Vaterlaus et al. [33] have identified already some years ago, that many users search for/get their motivation from sport-related posts. Overall, the profile of athletic sports offers simple but diverse possibilities for presentation on social media, e.g., short videos provide insights into the training and can represent support. Nevertheless, it is questionable to what extent the provided training information by these virtual professionals are reliable [34]. Further significant correlation could be found between resistance training and nutrition, as, beside training, nutrition is named to be one of the greatest influencing factors for successful sport performance [35]. Team sport was the only sport in our study that showed a significant correlation with the motive of social communication on social media. One reason for that could be that, beside few successful (elite) athletes in individual sports, the popularity and (digital) presence of sport teams is comparably high [36]. Notably, we could not find any correlations between the sport-related use of social media and the motive of self-staging. This finding is also in line with an earlier research by Carrotte et al. [37]. Thus, young adults mostly do not present themselves as sporty, but instead search for sport- and health-related information, social communication, as well as motivation and inspiration by fitness influencers. It can also be speculated that endurance sports are less important on social media than other forms of sport. In addition, the term endurance sport can be interpreted in many different ways, which is maybe why the respondents could not clearly assign the motives.

Taken together, the motives for sport-related use of social media mainly aim for the optimization of the body and allow the assumption that the participants may be concerned with conforming to an ideal of beauty and body shape. According to our hypotheses, especially resistance training showed a positive correlation with emulating sport-related beauty and body ideals. Most ideals of beauty and body shapes are primarily characterized by the build-up of muscles [38]. Similar results have been found in a study on the role of Instagram and Fitspiration images for muscular dysmorphic symptoms [39]. Especially the strength athletes responded to follow Fitspiration images, which in turn represent ideals of beauty and body shape. At the same time, as shown by the present study, many unconsciously know about the influence the different ideals of beauty and body shape on social media can have on one’s own body image.

### Sport-related social media usage and body image

On the one hand, our results showed that participants seem to be unsecure and obviously worry about their body image. On the other hand, the influence of social media on values of the VBD could also be demonstrated. These correlations strengthen our hypothesis about the relationship between sports, media activities and the tendentially negative effects on body image. These findings of the present study are in accordance with the study of Jiotsa et al. [40] indicating that the one of the age group of 18–25 years which have a conspicuous negative body image, strive for and compare themselves with presented beauty and body ideals on social media. As described, social media is being used more and more frequently for sport alongside actual training. In line, we found a significant correlation with a weak effect referring to this in the VBD group. There was also a smaller significant correlation between the VBD group and the following of sport-related content on social media. In turn, based on small effect size, a positive body image (VBD) can even be linked to posting one's own sport-related content. Taken together, although some persons benefit and are motivated by social media, their apparent positive inspiration is not to be equated with a stable positive body image. More than one third of participants experienced a negative influence of social media, of whom some occasionally felt poor health and social pressure. Respondents who were rather conspicuous in rejecting their body shape showed a strong correlation with dissatisfaction with one's own body when viewing posts on social media, as well as with the perception of social pressure when striving for perfect body image on social media. This supports the findings by Tiggemann and Zaccardo [41] as well as Raggatt et al. [42] who examined the initial decrease in body satisfaction and the emergence of social pressure. Not just the use of social media, but also the preoccupation with the perfect body has become part of the everyday life of many users [7]. The emulation of certain ideals of beauty and body shape is not a phenomenon of the digital age, but has always been present [43, 44]. The ideal conceptions classified by society are increasingly difficult to achieve, and thus, pose more risks for physical and mental health [45, 46]. If social media continues to be a platform for such risks, there is a need for educational advertising.

Taken together, although the current literature supports the assumption of both positive and negative effects of social media usage/content on young people, further research is needed to broaden the evidence. In our study, we were able to show that especially the engagement in athletic sports is highly related to the sport-related use of social media. This usage behavior and the presented (often fitspiration) content seems to have a great impact on the young generation. Aside the motivational and informational approach of social media, a negative influence or an uncertainty of the own body shape might occur. This finding is of particular interest as some could assume that the questioned target group should have adequate digital as well as sportive experience/understanding to deal constructively with the digital content. In general, our findings indicate that more educational advertising is needed, as the information shared/posted is hardly scientifically filtered. Many users implement, for example, the given training recommendations unknowingly and inexperienced. Thus, promoting media literacy is becoming even more relevant. Everyone should be able to deal critically with social media and inquire the available content. The *Body Positivity* movement is a first, important approach in this direction [20]. Beside critical evaluation, alternative body images are presented, so that this movement represents a kind of counter-current to distorted thin or athletic ideals. It is evident, that the resulting social pressure and effects on mental health will be a great challenge in the future to deal with personally and professionally. That is why the key message of Body Positivity, i.e., the satisfaction and appreciation of the own body, is getting of particular importance these days. Moreover, referring to its recent wide spread, the potentials of Body Positivity to establish a healthy and self-confident mindset especially in young adults should be further discussed and researched.

## Limitations

There are some constrains limiting our study: First, whilst the total sample size is quite representative, we decided to divide participants into two main age groups to avoid strongly deviating numbers of participants in smaller age subgroups, e.g., combined the age groups of 26–31 years and 31–35 years into the main age group of 26–35 years (*n* = 55). Due to the better fit to the definition of Digital Natives, only the participants in the age group 18–25 years (*n* = 230) were included in the inferential statistical analysis. In addition, the gender-specific analysis as well as the inferential statistical examination of the older age group were not further carried out as this would both exceed the scope of the present paper and need detailed gender- and age-specific discussion, but should be done in follow-up analyses. Second, in our study, we mostly related to photo- and video-based sporting social media content as they play a special role for the representation of ideals of beauty and body shape. Third, the questioned target group might be a limiting factor itself as its acquisition via local sports clubs and Instagram per se addressed individuals who are sportively active and have an affinity to social media. In turn, it can be assumed that hereby the sport-related use of social media automatically gains more importance than it probably might in other target groups. Additionally, the reliability of our results might be somewhat limited as our study is based on self-reported information which can be highly biased. At last, the direction of the connections between the sport-related use of social media and its influence on the individual’s body image emerged only superficially in our study, and needs to be examined more comprehensively. In addition, the application of a theory about social comparison and peer-group pressure could broaden overall data interpretation.

## Conclusion and future directions

The findings of the present study reveal the important role as well as the influence of social media on sport-related use and the resulting impact on young person’s body image. Social media do have the potential via sport-related content to motivate people to do sports, but at the same time, also some negative effects can be observed that should be prevented. Moreover, our results show that the influence of social media on the sport-related body image varies between person. The kind of sports as well as the individual approach to the own body seem to be the main moderating variables. In general, it is evident that the body images classified by society are increasingly difficult to achieve and thus, harbor more risks for physical and mental health. The attempt to achieve the given ideal of beauty is associated with effort and social pressure. I.e., as long as the fitness industry continues to grow and competition and success form the social benchmark, the social pressure to optimize one's own body will also remain. Notably, from a clinical perspective, this rise of social media and its content comprise a wide variety of dangers, from emerging social pressures to cyberbullying. Thus, especially education and sensibilization are needed to avoid the internalization of certain ideals of beauty and body shape in order to reduce the harmful effects associated with body dissatisfaction. Finally, tailored research is needed to assess the impact of sport-related use of social media more differentiated, and to establish, for example, suitable awareness interventions and user guidelines.

## Data Availability

The datasets used and/or analyzed during the current study are available from the corresponding author on reasonable request.

## Appendix A

See Tables 3, 4 and 5.

**Table 3:** Descriptive statistics of the sportive background of participants

	Answers	Total (N = 285)	Male (n = 101)	Female (n = 184)	Age group 18–25 years			Age group 26–36 years		
					Total (n = 230)	Male (n = 74)	Female (n = 156)	Total (n = 55)	Male (n = 27)	Female (n = 28)
Exercise frequency per week	0	13 (4.6%)	3 (3.0%)	10 (5.4%)	11 (4.8%)	3 (4.1%)	8 (5.1%)	2 (3.6%)	0 (0.0%)	2 (7.1%)
	1	28 (9.8%)	3 (3.0%)	25 (13.6%)	18 (7.8%)	2 (2.7%)	16 (10.3%)	10 (18.2%)	1 (3.7%)	9 (32.1%)
	2	44 (15.4%)	9 (8.9%)	35 (19.0%)	36 (15.7%)	7 (9.5%)	29 (18.6%)	8 (14.5%)	2 (7.4%)	6 (21.4%)
	3	64 (22.5%)	23 (22.8%)	41 (22.3%)	52 (22.6%)	15 (20.3%)	37 (23.7%)	12 (21.8%)	8 (29.6%)	4 (14.3%)
	4	50 (17.5%)	19 (18.8%)	31 (16.8%)	45 (19.6%)	15 (20.3%)	30 (19.2%)	5 (9.1%)	4 (14.8%)	1 (3.6%)
	5	43 (15.1%)	24 (23.8%)	19 (10.3%)	32 (13.9%)	15 (20.3%)	17 (10.9%)	11 (20.0%)	9 (33.3%)	2 (7.1%)
	6	29 (10.2%)	14 (13.9%)	15 (8.2%)	23 (10.0%)	12 (16.2%)	11 (7.1%)	6 (10.9%)	2 (7.4%)	4 (14.3%)
	7	14 (4.9%)	6 (5.9%)	8 (4.3%)	13 (5.7%)	5 (6.8%)	8 (5.1%)	1 (1.8%)	1 (3.7%)	0 (0.0%)
Training duration	< 30 min	27 (9.5%)	3 (3.0%)	24 (13.0%)	21 (9.1%)	2 (2.7%)	19 (12.2%)	6 (10.9%)	1 (3.7%)	5 (17.9%)
	31–45 min	47 (16.5%)	11 (10.9%)	36 (19.6%)	37 (16.1%)	9 (12.2%)	28 (17.9%)	10 (18.2%)	2 (7.4%)	8 (28.6%)
	46–60 min	84 (29.5%)	21 (20.8%)	63 (34.2%)	67 (29.1%)	13 (17.6%)	54 (34.6%)	17 (30.9%)	8 (29.6%)	9 (32.1%)
	61–90 min	101 (34.4%)	55 (54.5%)	46 (25.0%)	83 (36.1%)	42 (56.8%)	41 (26.3%)	18 (32.7%)	13 (48.1%)	5 (17.9%)
	> 90 min	26 (9.1%)	11 (10.9%)	15 (8.2%)	22 (9.6%)	8 (10.8%)	14 (9.0%)	4 (7.3%)	3 (11.1%)	1 (3.6%)
Main sport	Endurance sports	92 (32.3%)	26 (25.7%)	66 (35.9%)	73 (31.7%)	17 (23.0%)	56 (35.9%)	19 (34.5%)	9 (33.3%)	10 (35.7%)
	Resistance training/athletic sports	104 (36.5%)	48 (47.5%)	56 (30.4%)	87 (37.8%)	37 (50.0%)	50 (32.1%)	17 (30.9%)	11 (40.7%)	6 (21.4%)
	Team sports	39 (13.7%)	17 (16.8%)	22 (12.0%)	34 (14.8%)	14 (18.9%)	20 (12.8%)	5 (9.1%)	3 (11.1%)	2 (7.1%)
	Others	50 (17.5%)	10 (9.9%)	40 (21.7%)	36 (15.7%)	6 (8.1%)	30 (19.2%)	14 (25.5%)	4 (14.8%)	10 (35.7%)
Organizational form	Sports club	69 (24.2%)	22 (21.8%)	47 (25.5%)	62 (27.0%)	16 (21.6%)	46 (29.5%)	7 (12.7%)	6 (22.2%)	1 (3.6%)
	Commercial provider (e.g. fitness center)	87 (30.5%)	42 (41.6%)	44 (23.9%)	71 (30.9%)	35 (47.3%)	36 (23.1%)	16 (29.1%)	7 (25.9%)	9 (32.1%)
	Independent/self-organized	129 (45.3%)	37 (36.6%)	92 (50%)	97 (42.2%)	23 (31.1%)	74 (47.4%)	35 (63.6%)	14 (51.9%)	18 (64.3%)

**Table 4:** Chi-square tests of sports, motives and body image classification

	Endurance sports	Resistance training/athletic sports	Team sports	Others
Sport-related usage in general	χ^2^(1) = 1.57, *p* = 0.21, V = 0.08	χ^2^(1) = 5.86, *p* = 0.02*, V = 0.16	χ^2^(1) = 0.00, *p* = 1.00, V = 0.00	χ^2^(1) = 1.82, *p* = 0.18, V = 0.09
Training motivation	χ^2^(1) = 2.20, *p* = 0.14, V = 0.10	χ^2^(1) = 11.33, *p* < 0.001**, V = 0.22	χ^2^(1) = 4.25, *p* = 0.04*, V = 0.14	χ^2^(1) = 0.05, *p* = 0.83, V = 0.01
Training tips/information	χ^2^(1) = 2.04, *p* = 0.15, V = 0.09	χ^2^(1) = 6.96, *p* = 0.008**, V = 0.17	χ^2^(1) = 3.16, *p* = 0.08, V = 0.12	χ^2^(1) = 0.00, *p* = 0.97, V = 0.00
Training inspiration	χ^2^(1) = 0.01, *p* = 0.94, V = 0.01	χ^2^(1) = 0.17, *p* = 0.68, V = 0.03	χ^2^(1) = 0.00, *p* = 1.00, V = 0.00	χ^2^(1) = 0.46, *p* = 0.50, V = 0.04
Nutrition tips	χ^2^(1) = 0.23, *p* = 0.64, V = 0.03	χ^2^(1) = 5.59, *p* = 0.02*, V = 0.16	χ^2^(1) = 0.34, *p* = 0.56, V = 0.04	χ^2^(1) = 2.59, *p* = 0.11, V = 0.11
Social communication about sport	χ^2^(1) = 0.00, *p* = 1.00, V = 0.00	χ^2^(1) = 0.61, *p* = 0.44, V = 0.05	χ^2^(1) = 3.38, *p* = 0.07, V = 0.12	χ^2^(1) = 0.13, *p* = 0.72, V = 0.02
Sportive self-presentation	χ^2^(1) = 0.00, *p* = 1.00, V = 0.00	χ^2^(1) = 0.00, *p* = 1.00, V = 0.00	χ^2^(1) = 3.39, *p* = 0.08, V = 0.12	χ^2^(1) = 2.18, *p* = 0.17, V = 0.10
Marketing	χ^2^(1) = 0.00, *p* = 1.00, V = 0.00	χ^2^(1) = 3.75, *p* = 0.09, V = 0.13	χ^2^(1) = 0.02, *p* = 1.00, V = 0.01	χ^2^(1) = 5.51, *p* = 0.05, V = 0.15
Fan communication	χ^2^(1) = 2.36, *p* = 0.12, V = 0.10	χ^2^(1) = 0.07, *p* = 0.79, V = 0.02	χ^2^(1) = 0.97, *p* = 0.33, V = 0.06	χ^2^(1) = 0.08, *p* = 0.77, V = 0.02
Emulation of beauty and body ideals	χ^2^(4) = 6.18, *p* = 0.19, V = 0.16	χ^2^(4) = 22.05, *p* < 0.001**, V = 0.31	χ^2^(4) = 5.53, *p* = 0.24, V = 0.16	χ^2^(4) = 4.75, *p* = 0.31, V = 0.14
Rejecting body image (RBI)	χ^2^(2) = 1.30, *p* = 0.52, V = 0.08	χ^2^(2) = 0.30, *p* = 0.86, V = 0.04	χ^2^(2) = 0.27, *p* = 0.87, V = 0.03	χ^2^(2) = 0.17, *p* = 0.92, V = 0.03
Vital body dynamics (VBD)	χ^2^(2) = 1.38, *p* = 0.50, V = 0.08	χ^2^(2) = 2.02, *p* = 0.36, V = 0.09	χ^2^(2) = 0.92, *p* = 0.63, V = 0.06	χ^2^(2) = 1.61, *p* = 0.45, V = 0.08

*N* = 230, **p* < 0.05, ***p* < 0.01

**Table 5:** Spearman-correlations of body image classifications and sport-related usage of social media

Item	Rejecting body image (RBI)	Vital body dynamics (VBD)
Emulation of sport-related ideals of beauty and body shape	r = 0.63, *p* = 0.001**	r = 0.03, *p* = 0.646
Sport-related usage of social media off training	r = 0.51, *p* = 0.433	r = 0.23, *p* = 0.001**
Actively following sport-related content/fitness influencers	r = 0.10, *p* = 0.118	r = 0.20, *p* = 0.002**
Posting sport-related content	r = 0.07, *p* = 0.229	r = 0.18, *p* = 0.007**
Increasing dissatisfaction towards the own body when following sport-related content	r = 0.59, *p* = 0.001**	r = -0.13, *p* = 0.057
Social pressure to optimize/have the perfect body	r = 0.50, *p* = 0.001**	r = -0.68, *p* = 0.301
Comparison of own body with virtual presented body ideals	r = 0.48, *p* = 0.001**	r = -0.08, *p* = 0.209

*N* = 230, ***p* < 0.01

## Appendix B

See Fig. 1.

## Abbreviations

FKB-20: Body image questionnaire
RBI: Rejecting body image
VBD: Vital body dynamics
